# Botulinum Toxin in Abdominal Wall Reconstruction: A PropensityMatched Cohort of 5,000 Patients with a 5-Year Follow-up

**DOI:** 10.1093/asjof/ojag138.008

**Published:** 2026-07-24

**Authors:** Yousef Tanas, Stephen Chen, Philong Nguyen, Joshua Wang, Dora Lena Fedorcsak, Tue Dinh

**Affiliations:** Institute for Reconstructive Surgery, Houston Methodist Hospital, Weill Cornell Medicine, Houston, Texas, USA; Baylor College of Medicine, Houston, Texas, USA; University of Texas Medical Branch, Galveston, Texas, USA; University of Texas Medical Branch, Galveston, Texas, USA; Semmelweis University, Budapest, Hungary; Institute for Reconstructive Surgery, Houston Methodist Hospital, Weill Cornell Medicine, Houston, Texas, USA

## Abstract

**Goals/Purpose:**

Preoperative botulinum toxin A (BTX) is widely adopted to facilitate midline fascial closure during complex abdominal wall reconstruction (AWR), yet its long-term safety and effectiveness remain uncertain. This study evaluates 5-year outcomes after AWR performed with versus without BTX, quantifying effects on hernia recurrence, woundrelated complications, and reoperation, and identifying the net clinical trade-offs relevant to reconstructive and body-contouring decision-making.

A retrospective cohort analysis was conducted using the TriNetX National Health Research Network. Adults undergoing AWR were identified by ICD-10/CPT codes. Exposure was receipt of BTX prior to AWR (BTX-AWR) versus no BTX (No-BTX-AWR). Outcomes were assessed cumulatively at 6 months, 1 year, 3 years, and 5 years: any infection, wound disruption/dehiscence, seroma, hematoma, surgical site infection (SSI), reoperation, and hernia recurrence. Propensity-score matching (1:1, nearest neighbor, caliper 0.2 SD of the logit) balanced groups on demographics, comorbidities (obesity, diabetes, smoking, COPD), hernia-related risk markers, and perioperative factors available in the EHR. Balance was verified with standardized mean differences (<0.1). Risk estimates (absolute risk, risk difference, risk ratio/odds ratio) and 95% CIs were calculated with two-sided α=0.05. Because dosing/timing of BTX and operative techniques are not standardized in EHR data, these were handled as unmeasured confounders and addressed in limitations. The study used de-identified data and did not require IRB review per network policy.

**Results/Complications:**

Before matching, 2,584 BTX-AWR and 343,589 No-BTX-AWR cases were identified. After matching, cohort sizes per time point were: 6 months (n=2,255 per group), 1 year (n=2,508), 3 years (n=2,279), and 5 years (n=2,432). BTX-AWR had consistently higher cumulative infection risk (6 mo: 14.4% vs 10.5%, p<0.001; 1 yr: 17.6% vs 11.9%, p<0.001; 3 yr: 22.6% vs 15.3%, p<0.001; 5 yr: 25.1% vs 16.8%, p<0.001). Wound disruption/dehiscence was higher with BTX-AWR at all intervals (6 mo: 8.7% vs 4.7%, p<0.001; 1 yr: 9.4% vs 5.5%, p<0.001; 3 yr: 10.8% vs 6.6%, p<0.001; 5 yr: 11.3% vs 5.0%, p<0.001). Seroma was initially comparable early but higher with BTX-AWR later (3 yr: 2.6% vs 1.2%, p=0.001; 5 yr: 2.8% vs 1.7%, p=0.009). Both Hematoma and SSI were higher in BTX-AWR throughout follow-up (e.g., hematoma 6 mo 1.3% vs 0.5%, p=0.003; SSI 6 mo 3.9% vs 2.4%, p=0.002; differences persisted through 5 years). Reoperation was elevated with BTX-AWR at every time point, increasing over time (6 mo: 10.7% vs 7.8%, p=0.001; 5 yr: 20.3% vs 13.4%, p<0.001). Hernia recurrence was lower with BTX-AWR at 6 months but not thereafter (6 mo: 32.1% vs 35.6%, p=0.013; 1 yr: 36.3% vs 37.7%, p=0.32; 3 yr: 41.6% vs 43.0%, p=0.323; 5 yr: 42.6% vs 44.8%, p=0.112).

Interpretation of complications: The BTX-AWR group demonstrated a clear early signal toward improved fascial closure durability (lower short-term recurrence), offset by higher risks of infection-related events, wound disruption, and reoperation extending to 5 years. These patterns suggest (1) possible confounding by indication (BTX preferentially used in more complex, high-risk hernias), and (2) that gains in early closure may incur downstream wound/revision trade-offs, particularly when perioperative optimization and infectionprevention bundles are not standardized.

**Conclusion:**

In a large, nationally representative matched cohort with 5-year follow-up, preoperative BTX use in AWR was associated with lower short-term hernia recurrence but higher risks of infection, wound disruption, SSI/hematoma, and reoperation across all time points. For reconstructive and aesthetic surgeons managing patients with diastasis or concomitant ventral hernias, BTX may be best reserved for carefully selected complex defects where early closure is otherwise unlikely. Shared decision-making should emphasize the trade-off between early closure benefits and higher complication/reoperation risks. Standardized protocols for patient optimization, BTX timing/dose, and infection prevention may mitigate unfavorable signals. Prospective studies—ideally randomized or protocolized multicenter cohorts—are needed to establish causality and refine candidate selection criteria.

